# Mycetoma Medical Therapy

**DOI:** 10.1371/journal.pntd.0003218

**Published:** 2014-10-16

**Authors:** Oliverio Welsh, Hail Mater Al-Abdely, Mario Cesar Salinas-Carmona, Ahmed Hassan Fahal

**Affiliations:** 1 Department of Dermatology, Dr. Jose E. Gonzalez University Hospital, Universidad Autónoma de Nuevo León, Monterrey, Nuevo León, Mexico; 2 Section of Infectious Diseases, Department of Medicine, King Faisal Specialist Hospital and Research Centre, Riyadh, Saudi Arabia; 3 Department of Immunology, Faculty of Medicine, Universidad Autónoma de Nuevo León, Monterrey, Nuevo León, Mexico; 4 The Mycetoma Research Centre, University of Khartoum, Khartoum, Sudan; University of Tennessee, United States of America

## Abstract

Medical treatment of mycetoma depends on its fungal or bacterial etiology. Clinically, these entities share similar features that can confuse diagnosis, causing a lack of therapeutic response due to inappropriate treatment. This review evaluates the response to available antimicrobial agents in actinomycetoma and the current status of antifungal drugs for treatment of eumycetoma.

## Introduction

Mycetoma is a potentially serious, devastating, chronic, inflammatory disease caused by aerobic actinomycetic bacteria (actinomycetoma) or fungi (eumycetoma). The worldwide incidence of actinomycetoma and eumycetoma varies from country to country and region to region, but this infection is predominant in countries that are located between 30°N and 15°S. Most cases of mycetoma occur in Sudan, Venezuela, Mexico, and India. Sudan has the highest incidence of eumycetoma (up to 70%). In Mexico, actinomycetoma predominates in about 97% of cases [1]. The clinical picture of both infections is quite similar. To achieve cure, it is important to define the fungal or bacterial etiology because treatment for each is completely different. Actinomycetoma is currently treated with antibiotics, which can be used alone or in different combinations depending on the severity, dissemination, and location of the disease. Medical cure is generally achieved if the patient is properly treated [2]. In contrast, treatment of eumycetoma consists of antifungals and surgical excision [3]. Medical cure is more difficult to obtain and the extension and location of the disease may lead to chronic progressive lesions that often lead to amputation [4]. However, in both forms of mycetoma, prolonged treatment is needed. Our objective is to present the current status of medical therapy of mycetoma and the best antibiotic and antifungal options available for its management.

## Methods

Most of the literature we reviewed related to treatment was based on case reports and in vitro and in vivo studies of drug susceptibilities. We assessed and collected leading articles on drugs that are available and have been successfully used in patients with actinomycetoma and eumycetoma. Adverse effects, drug interactions, dose, and duration of treatment were considered. We appraised publications most useful for readers interested in the medical management of this neglected disease.

## Actinomycetoma

### Causative agents

Three genera (*Nocardia*, *Streptomyces*, and *Actinomadura*) comprise the most frequent causative agents of actinomycetoma. Etiological agents of actinomycetoma include *Nocardia brasiliensis*, *N. asteroides*, *N. caviae*, *N. farcinica*, *N. transvalensis*, *N. dassonvillei*, *N. mexicana*, *N. veterana*, *Actinomadura madurae*, *A. pelletieri*, *A. latina*, *Streptomyces somaliensis*, and *S. sudanensis*. New species of *Nocardia* that have been reported to cause actinomycetoma are *N. harenae* and *N. takedensis* [5], [6].

### In vitro susceptibility data

Studies of sensitivity of *N. brasiliensis* to different antimicrobials and antibiotics have been reported in vitro and in vivo [7]–[10]. A study by Gomez et al. demonstrated that the best inhibitory effect occurs with aminoglycosides (100% susceptibility to amikacin, gentamicin, isepamicin, netilmicin, and tobramycin); however, all strains were resistant to streptomycin and kanamycin. *Nocardia* strains were also susceptible to linezolid in 100% of cases and to sulfonamides (trimethoprim-sulfamethoxazole) in 83% [10]. Amoxicillin-clavulanate showed an inhibitory effect of 97%. Other oxazolidinones have been evaluated in the laboratory and found effective for future treatment in cases that could be resistant to other antimicrobials [11].

The susceptibility of 30 strains of *N. brasiliensis* isolated from patients with actinomycetoma was determined using econazole, imipenem, and meropenem, both alone and combined with clavulanic acid. MIC_50_ and MIC_90_ values for econazole were 2 and 4 µg/ml, respectively. For imipenem, values were 64 and 64 µg/ml, respectively. Only seven isolates had a minimum inhibitory concentration (MIC) of 2 µg/ml. Regarding meropenem, MIC values were 2 and 8 µg/ml with 16 out of 30 isolates exhibiting an MIC of 2 µg/ml. The addition of clavulanic acid to the carbapenems did not significantly change MIC values [8]. Because of the cost of carbapenems, it is necessary to determine if the isolated strain is susceptible to these antibiotics.

Molecular studies have also been useful to identify the species of the infecting organism with greater specificity [7], [9].

### Animal models

Animal models have been successfully used to study the pathogenic mechanism of actinomycetoma and the therapeutic efficacy of diverse antimicrobials. Experimental *N. brasiliensis* actinomycetoma infection was induced by inoculation in the footpad of immunocompetent and athymic nude homozygous and heterozygous Lewis rats. Classic actinomycetoma lesions occurred in the infected foot of the immunocompetent rats. After 20–25 days, the lesions began to heal. A more active infection was found in homozygous athymic rats, and some animals died because of dissemination of the infection with organ involvement. Histopathological examination showed an infiltrate mainly of polymorphonuclear cells; after 20 days, the infiltrate was composed mostly of histiocytes, lymphocytes, fibroblasts, and Langhans cells. The presence of grains was observed after 15 days in heterozygote Lewis rats, but not in homozygous nude rats [12].

Studies in BALB/c mice have allowed analysis of the inflammatory mechanisms and therapeutic effect of diverse antimicrobials [13]. Since combination therapy seems to work better for actinomycetoma, amikacin, SXT, amoxicillin-clavulanic acid, and linezolid were analyzed to determine the effect of drug combinations on *N. brasiliensis* [10]. Some of the combinations tested, particularly amoxicillin-clavulanic acid in combination with linezolid, showed synergistic activity.

Because of the ethical and patient selection difficulties in carrying out prospective clinical trials in endemic areas, the animal model becomes a useful alternative to determine which antimicrobials could be therapeutically effective in human infection.

### Clinical data

Actinomycetoma frequently affects the feet and legs; in Mexico, the back is the second most frequent location affected, but different body parts may also be affected. The infection involves subcutaneous tissues and can disseminate to underlying structures, such as bone and organs. It is characterized by a painless, firm mass with nodules, abscesses, fistulae, and draining sinuses discharging a syrup-like filamentous exudate that contains aerobic grains of the causative organism. The differential diagnosis includes other bacterial infections causing osteomyelitis, tuberculosis, other mycobacterial infections, subcutaneous and systemic mycoses, and neoplasia [2].

In contrast to eumycetoma, in actinomycetoma, surgery is seldom used. Most cases respond to medical therapy, although some require prolonged administration of antimicrobial combinations (for weeks or months) (Table 1) [2].

**Table 1:** In vitro susceptibility, clinical efficacy, and dose of current antibiotics for actinomycetoma and antifungal agents against *M. mycetomatis*.

Antibiotics	In vitro	Human infection	Dose
Sulfonamides DDS (4,4 diaminodiphenyl-sulfone)	No data	Effective	100–200 mg/day single dose
Trimethoprim-Sulfamethoxazole (TS)	Active	Effective	8 mg/40 mg
Amikacin sulphate-TS	Active	Effective	Amikacin: 15 mg kg/day IM or IV in two daily doses; TS as above
Netilmicin-TS	Active	Effective	Netilmicin 300 mg/day IM single dose; TS as above
Minocycline	Active	Effective in 70%	200 mg/day PO in divided dose
Amoxicillin-clavulanate	Active	Effective	500 mg/125 mg PO; tid for 3 to 6 months
Linezolid	Active	Effective	600 mg PO twice daily
Fosfomycin	Active	Effective	100–200 mg/kg/day q6-8 h IV or PO in 21-day cycles.
Imipenem	Active depending on the strain	Effective depending on the strain	500 mg IV q8 hours; not to exceed 50 mg/kg/day or 4 g/day
Meropenem	Active	Effective	500 mg IV q8 hr; not to exceed 2 g IV daily
Rifampicin	Active depending on the strain	Effective depending on the strain	10 mg/kg/day PO
Moxifloxacin	Active	Effective	400 mg/day IV or PO
**Antifungal agents**			
Amphotericin B	Moderate activity	Not effective	
Fluconazole	Limited activity	Not effective	
Ketoconazole	Active	Variable efficacy	400–800 mg
Itraconazole	Active	Variable efficacy	200–400 mg
Voriconazole	Active	Effective in few case reports	200 mg
Posaconazole	Active	Effective in few cases	
Isavuconazole	Active	No data	
Echinocandins	Not active	No data	
Terbinafine	Moderate activity	No data	

Possible drug interactions, history of drug allergies, and co-morbidities should be analyzed in all drugs.

IM, intramuscularly; IV, intravenous; PO, orally; tid, three times daily.

### Current treatment of actinomycetoma

Effective medical treatment of actinomycetoma began in the early 1940s and 1950s with the use of sulfonamides and diamino diphenyl sulphone (DDS), achieving cure in some cases. In the 1960s, trimethoprim-sulfamethoxazole (TS) became standard treatment for actinomycetoma. This drug was given for 3–4 months, and in some cases, for longer periods. Other antibiotics, such as streptomycin, isoniazid, rifampin, and minocycline, have been added in isolated cases that did not respond to TS [14], [15]. There are no comparative studies of the efficacy of these drugs in combination with TS.

Treatment with TS continued in the 1970s [16]. In 1982, a case of severe actinomycetoma successfully treated with a combination of amikacin sulphate and TS was reported [17]. The patient was a 19-year-old man with multiple lesions and ulcers on his chest wall and with pulmonary involvement accompanied with malaise. The causative organism was identified as *N. brasiliensis* and was isolated from skin lesions, pleural effusion fluid, and blood. Skin biopsy from the affected site revealed multiple granulomas and *Nocardia* grains. The colony was sent to the American Type Culture Collection (ATCC) reference database for characterization and further study. The strain was named HUJEG-1 (*N. brasiliensis* ATCC 700358). The complete sequence of this strain was achieved in 2012 [18].

Because of the infection severity and its dissemination, the authors sought treatment alternatives and selected amikacin sulphate because of its in vitro inhibitory activity against *N. asteroides*. This drug was combined with TS and given as follows: amikacin 15 mg/kg/day intramuscularly (IM) divided into two daily doses for 3 weeks simultaneously with TS 8/40 mg/kg/day orally for 5 weeks. At the end of this time, the patient obtained an improvement of 90% and all pulmonary lesions disappeared. He was released from the hospital and did not return for evaluation, nor did he continue any treatment. About a year later, the patient was seen, and he was completely cured.

The results obtained in this patient led to a prospective study with this combination scheme in severe cases of actinomycetic mycetoma that did not respond to TS alone [19]. Up to 1989, a total of 25 patients unresponsive to previous therapy or with extensive involvement and/or risk of dissemination to underlying organs were treated. Depending on the severity and extension of disease, some patients were treated as inpatients and others as outpatients. The combination was administered in 5-week cycles (3 weeks of amikacin sulphate intramuscularly together with 5 weeks of oral TS). Audiometry and creatinine clearance were performed before and after each cycle of amikacin sulphate. Depending on the clinical response, this cycle of treatment was consecutively repeated for up to four cycles. All patients in this group were cured except for one who after 3 months developed a recurrence that required further treatment [20].

**Figure 1:**
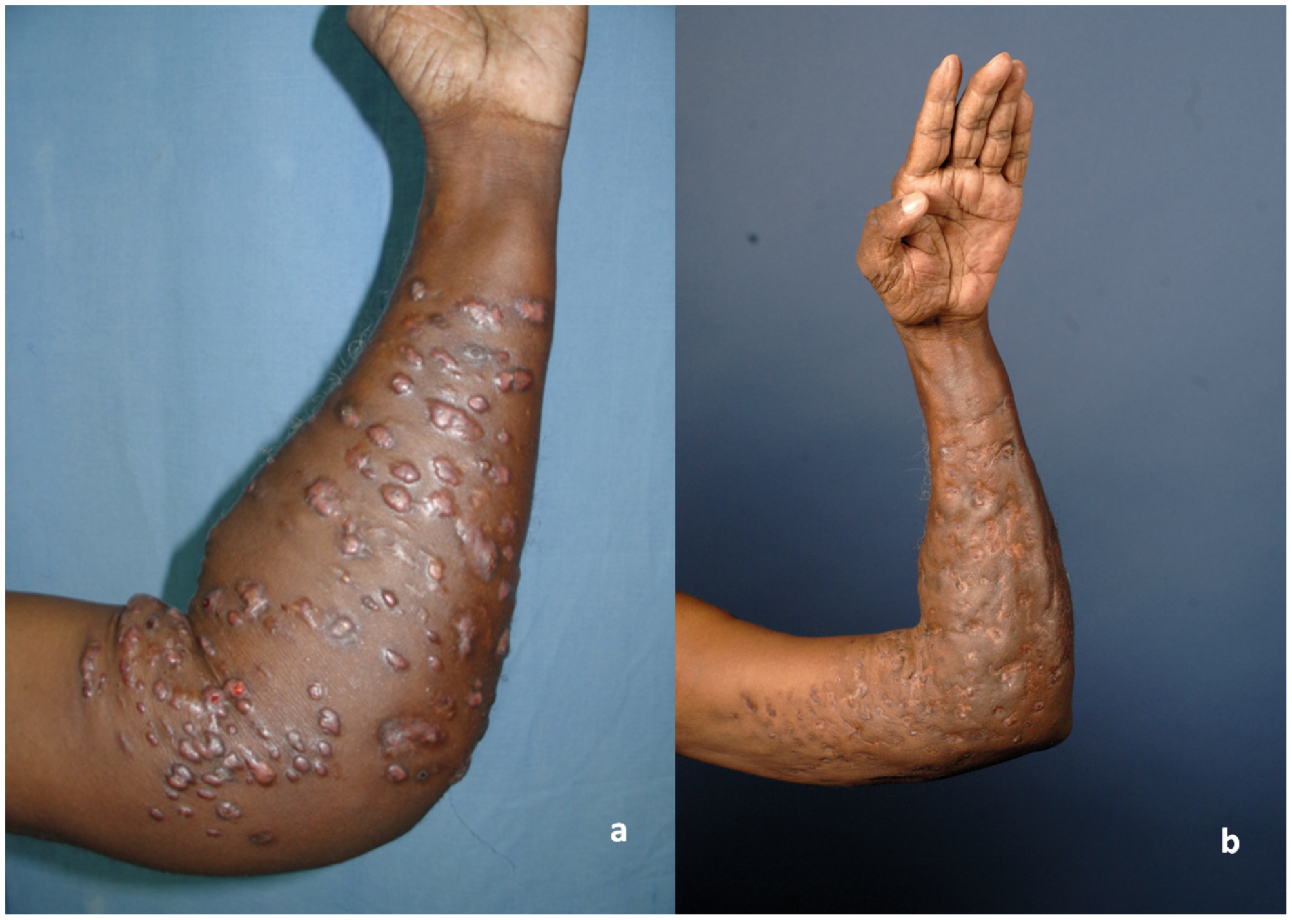
Clinical outcome of patient with actinomycetoma treated with amikacin and trimethoprim/sulfamethoxazole, before (a) and after (b) therapy.

To date, the response to this combination has been encouraging (see Figure 1) [2], achieving a cure rate of about 90% (56 patients). Twenty percent of these patients developed minimal or moderate auditory changes detected by audiometry. In one patient, it was severe and detected clinically. In three patients, the medication was stopped; in one because of drug allergy, another due to development of bacterial resistance, and in a third because of recurrence 2 years after remission. Treatment in this patient was continued with a combination of TS, moxifloxacin, netilmicin, and imipenem, and he was cured.

Different antibiotics have been assayed in vitro, ex vivo, and in experimental *N. brasiliensis* actinomycetomas to find treatment alternatives. Among these are TS, amikacin, other aminoglycosides, amoxicillin-clavulanic acid, minocycline, moxifloxacin, linezolid, and carbapenems [7], [10], [11], [21]–[23]. Most of the recalcitrant cases in patients have responded well to amikacin/TS and only a few had further treatment with imipenem and/or carbapenem [2].

### Eumycetoma treatment in Mexico

Eumycetoma occurs in Mexico in 3.48% of cases [1]. Treatment is based on prolonged administration of imidazoles such as itraconazole, alone or combined with terbinafine [24]. Posaconazole and voriconazole are available but are expensive, and their therapeutic efficacy has not been assayed. Amphotericin B is rarely used. The combination of medical and surgical treatment is the usual management of this fungal infection.

### Adverse effects

Amikacin sulphate or the administration of any aminoglycoside requires close clinical observation with audiometry and renal function tests every 3 to 5 weeks to detect auditory and nephrotoxicity and adjust dosing accordingly. Loop diuretics should be avoided with amikacin sulphate because of potential cochlear damage [25]. Cephalothin may increase the risk of aminoglycoside nephrotoxicity [26].

Side effects of other currently used antimicrobials should be considered. Other nephrotoxic antibiotics (vancomycin, meropenem) in combination with aminoglycosides must be avoided because they can increase potential nephrotoxicity. Dosing must be adjusted for renal function and for haemofiltration.

Imipenem and meropenem should not be prescribed to patients who are allergic to penicillin and other β-lactam antibiotics [27].

Mild adverse events with linezolid are diarrhea, headache, and nausea. An important adverse effect, myelosuppression, has been reported with high and prolonged doses. Blood parameters return to normal after discontinuing the drug and a complete blood count must be performed weekly. Neurological symptoms, such as peripheral and optic neuropathy, can develop [28], [29].

Trimethoprim/sulfamethoxazole can produce dermatologic reactions, usually due to hypersensitivity, such as rash, pruritis, photosensitivity reactions, exfoliative dermatitis, erythema nodosum, and haemolytic anemia. Patients should be well hydrated and with an alkaline urine because sulfamethoxazole may cause sulfa crystalluria. Gastrointestinal and other haematologic, renal, hepatic, metabolic, and nervous system effects should be evaluated before and during drug administration [30]. Antibiotics and doses that are currently available and can be used for treatment of actinomycetoma are shown in Table 1.

## Eumycetoma

### Causative agents

Causative agents of eumycetoma are classified into those that produce black grains and those that produce white or grayish grains: *Acremonium falciforme*, *Acr. kiliense*, *Acr. recifei*, *Aspergillus flavus*, *Asp. nidulans*, *Cladophialophora bantiana*, *Cochliobolus spicifer*, *Corynespora cassicola*, *Curvularia geniculata*, *Cur. lunata*, *Cylindrocarpon cyanescens*, *Cyl. destructans*, *Drechslera rostrata*, *Exophiala jeanselmei*, *Exserohilum rostratum*, *Fusarium* spp., *Fusarium moniliforme*, *F. oxysporum*, *F. solani*, *Leptosphaeria senegalensis*, *L. tompkinsii*, *Madurella mycetomatis*, *M. grisea*, *M. fahalii*, *Neotestudina rosatii*, *Phaeoacremonium krajdenii*, *Phialophora cyanescens*, *Plenodomus avramii*, *Polycytella hominis*, *Pseudallescheria boydii*, *Pseudochaetosphaeronema larense*, *Pyrenochaeta mackinnonii*, *P. romeroi*, and *Scedosporium apiospermum*. Four etiological agents cause more than 90% of the eumycetomas worldwide. These are *M. mycetomatis, M. grisea, Pseudosporium boydii, and L. senegalensis* [31], [32].

### In vitro susceptibility data

Several in vitro studies have been conducted on fungal organisms that commonly cause eumycetoma. Most of the studies were of *M. mycetomatis* and *Scedosporium boydii complex (Sc. apiospermum, Sc. boydii, Sc. aurantiacum)* and a few agents of phaeohyphomycosis that can cause mycetoma such as *Exophiala jeanselmei* [33]–[40]. Almost no data have been reported for *Falciformispora senegalensis* (synonym: *L. senegalensis*) and *Medicopsis romeroi* (synonym: *P. romeroi*) [41]. van de Sande and colleagues have conducted several studies on *M. mycetomatis* susceptibility to many of the currently available antifungals. In vitro testing was done by known methods for filamentous fungi. These methods included the CLSI broth dilution method and the colorimetric Sensititre YeastOne test, as well as the viability-based XTT test. The three methods were compared by testing 36 isolates of *M. mycetomatis* against six antifungals: amphotericin B, ketoconazole, fluconazole, itraconazole, voriconazole, and 5-flucytosine [34]. The Sensititre test was comparable to the CLSI method but produced lower MICs when compared to the viability-based XTT test. This was more obvious with the azoles. The most active antifungals, in vitro, were ketoconazole and the extended-spectrum triazoles, itraconazole and voriconazole. Amphotericin B had a median MIC of 1 µg/mL, while fluconazole had limited activity, and 5-flucytosine had no activity against *M. mycetomatis*. The echinocandins, caspofungin, micafungin, and anidulafungin showed no activity in vitro against 17 isolates of *M. mycetomatis* utilizing the XTT method [35]. However, another study of three isolates of *M. mycetomatis* against anidulafungin using the CLSI method had an MIC of 1 µg/mL [42].

The role of melanin in fungal resistance to antifungals in *M. mycetomatis* is not clear. One study has shown a several-fold increase in MICs to ketoconazole and itraconazole with the addition of melanin to the culture media [43]. Posaconazole and isavuconazole have good activity against *M. mycetomatis*, *Sc. apiospermum* and *E. jeanselmei*. MICs against *M. mycetomatis* were in the range of 0.016 to 0.25 µg/mL [36], [44].

The allylamine antifungal, terbinafine, showed moderate activity against *M. mycetomatis* and variable activity against *Sc. apiospermum* [36], [45]–[47]. Several in vitro studies have indicated low MICs for itraconazole and voriconazole to several strains of *Sc. apiospermum* [37], [39], [45], [48]. Several agents of phaeohyphomycosis, including *E. jeanselmei*, are susceptible in vitro to itraconazole, voriconazole, and posaconazole [39], [40], [49], [50].

Standardization of the testing methodology is required to be able to compare evidence from various studies against filamentous fungi. Many of these fungi do not sporulate or may have variable sporulation within different strains of the same species. Using a hyphal inoculum may not be similar to a conidial inoculum, and therefore gives different results [51]. The extended spectrum triazoles and ketoconazole have the best activity against *M. mycetomatis*, while ketoconazole has limited or variable activity against *Sc. boydii* complex and phaeohyphomycetes.

### Animal models

Few experimental animal models on the development of *M. mycetomatis* eumycetoma infection have been published. Data regarding experimental fungal infections that can cause mycetoma come from a few successful models (athymic mice, BALB/c mice and goat); however, none of these have evaluated the therapeutic effect of antifungals on *M. mycetomatis* infection [52], [53]. Animal models for therapy of infections due to *S. boydii* complex and agents of phaeohyphomycosis are several, including murine, rat, and guinea pig [54], [55]. *Sc. apiospermum* experimental infection in mouse and guinea pig has shown the efficacy of voriconazole and posaconazole [56]–[58]. A high dose of posaconazole was required for efficacy in a murine model of disseminated *Sc. apiospermum* infection, while itraconazole was not effective [57]. Higher MIC to voriconazole correlated with failure of experimental therapy in one study [54]. Several phaeohyphomycetes, including *Exophiala* species, were responsive to itraconazole and posaconazole in an experimental murine infection [59]. Posaconazole demonstrated the best activity in these animal studies [60], [61].

### Clinical data

Published studies indicate the need for combined medical and surgical therapy to achieve success in fungal mycetoma. Factors that determine therapy outcome include extent of tissue and bone involvement, site of the disease, and antifungal therapy. It is not yet clear if the extent of surgical debridement and the type and duration of antifungal therapy alter outcome [4]. However, near complete surgical excision and prolonged antifungal therapy is more likely to succeed. Timing of surgery in relation to antifungal therapy is not well established. One prospective study indicates that medical therapy may limit the disease and make complete excision of the lesions more feasible [3].

### Current treatment of eumycetoma

As neglected diseases, mycetoma in general and fungal mycetoma in particular have received little attention in the development of specific therapeutics. All currently used drugs against causative agents of eumycetoma were developed and studied with other, more common fungi [62]. For several decades, systemic antifungal therapy has been limited to a few drugs that are potentially toxic and delivered parenterally. Amphotericin B deoxycholate was widely used despite its toxicity. However, a wide range of antifungal agents have been approved and marketed for various fungal infections [62]. These include less toxic lipid formulations of amphotericin B, second and third generation azoles, terbinafine and echinocandins. The newer azoles are broad-spectrum and oral, with good bioavailability and low toxicity [63]. These agents are particularly attractive for prolonged outpatient therapy, which is typically needed in a chronic fungal infection such as eumycetoma; however, there are limited in vitro and in vivo studies. Clinical data are almost exclusively from case reports and a small number of case series. Prospective clinical studies are needed to evaluate the therapeutic potential of these antifungals.

Amphotericin B was the only systemic antifungal available for almost three decades. It was not widely used for eumycetoma because of significant toxicity and the need to be given parenterally for prolonged periods. Lipid-associated amphotericin B was tried at the Mycetoma Research Centre in Sudan in four patients, but the results were disappointing. One patient had acute renal failure; treatment was stopped and he recovered. The other three patients had courses of 6 weeks duration with no dramatic response, and viable organisms were cultured from the lesions.

The imidazole ketoconazole, introduced in the early 1980s, was a breakthrough in systemic antifungal therapy. It is active against *Candida* and several other fungi and can be administered orally. Mahgoub and Gumaa published their experience with ketoconazole therapy in 13 patients with mycetoma due to *M. mycetomatis* from Saudi Arabia and the Sudan. Doses ranged from 200 to 400 mg daily, and the therapeutic response was variable: ten patients had a good response and three did not [64]. The follow-up period was short in half the patients; therefore, the frequency of relapse could not be determined. Afterwards, reports of variable responses with ketoconazole in different parts of the world were published [65], [66]. In a report from India, six out of ten patients were reported cured of fungal mycetoma after prolonged therapy with ketoconazole (8 to 24 months) [66]. However, recently, the use of ketoconazole has been limited by the United States Food and Drug Administration and the European Medicines Agency (EMA) due to its hepatic and adrenal toxicity. Ketoconazole should not be used as first-line treatment. It is recommended only for the treatment of certain life-threatening fungal infections (endemic mycoses) when alternative antifungal therapies are not available or tolerated [67]. Fluconazole is not an effective therapy for eumycetoma and is currently not used for treatment [68].

Itraconazole was released in the early 1990s and became the most commonly used drug for the treatment of eumycetoma in places where it was affordable. The bioavailability of itraconazole is variable, and absorption is related to stomach acidity and food. Reports indicate a clinical response to itraconazole in patients with eumycetoma [3], [69]–[71]. These are mostly retrospective case series or case reports that suggest a variable response. In one prospective non-comparative study of medical therapy with itraconazole for 12 months followed by surgical excision in 13 subjects, most patients had a favorable outcome [3].

Limited data is available on the new classes of antifungals (Table 1). Few case reports show a good response to voriconazole [72], [73]. Treatment with posaconazole was successful in one case and stable in another due to *M. mycetomatis*, three cases due to *M. grisea* were successful, and one case due to *Sc. apiospermum* had a partial improvement [74]. Duration of therapy and extent of surgical debridement was variable among these cases.

Terbinafine given in a high dose was successful in a few cases of eumycetoma and in two cases of disseminated *E. jeanselmei* infection [75]–[76]. In a study of 23 patients, terbinafine at a high dose of 500 mg twice daily for 24–48 weeks resulted in 25% cure and 55% improvement of patients [77]. Terbinafine was not effective in deep-seated infections due to *Sc. apiospermum* [78], [79]. Both voriconazole and posaconazole were reported to be efficacious in disseminated infections due to *Sc. apiospermum* [80]–[85]. There are no clinical data on the efficacy of echinocandins or the investigational triazole isavuconazole.

### Adverse effects

It is important to evaluate possible drug interactions of azoles. Antacids may reduce their absorption, and azoles may cause edema when calcium channel inhibitors are used. Hypoglycemia may occur with concomitant use of sulfonylureas. Azoles may increase plasma concentrations of tacrolimus and cyclosporine at high doses and they can also increase digoxin levels and plasma levels of midazolam and triazolam. Rhabdomyolysis has been reported with cholesterol-lowering drugs (lovastatin and simvastatin) and severe cardiac arrhythmias and possible sudden death with cisapride. Co-administration with phenytoin, rifampin, and H2 receptor antagonists causes a reduction in azole plasma levels. Imidazoles can increase the anticoagulant effect of warfarin. Simultaneous treatment with warfarin and imidazoles should be carefully monitored. The patient must avoid alcohol consumption, and liver function should be periodically monitored [24].

Notable adverse effects of ketoconazole are hepatotoxicity, gynecomastia, lip dryness and ulceration, skin hyperpigmentation, and decreased libido. Itraconazole is contraindicated in patients with evidence of ventricular dysfunction such as congestive heart failure or a history of congestive heart failure [86].

Posaconazole can cause fever, diarrhea, nausea, vomiting, and headache. Other adverse events include hypokalemia, rash, thrombocytopenia, and abdominal pain. Liver function tests should be performed at baseline and throughout therapy to monitor possible liver damage. Treatment should be discontinued if serious liver abnormalities occur. Rare serious adverse events are hemolytic uremic syndrome, thrombotic thrombocytopenic purpura, pulmonary embolus, adrenal insufficiency, and allergic and/or hypersensitivity reactions. Prolongation of the QT interval may be seen [87].

Common side effects of voriconazole are visual alterations, fever, rash, vomiting, nausea and diarrhea, headache, sepsis, peripheral edema, abdominal pain, and respiratory disorders [24].

## Conclusion

The therapeutic outcome of mycetoma depends on the bacterial or fungal etiology of the infection; factors such as the infecting agent; and the patient's social and economic status, cultural background, nutrition, therapeutic compliance, and resistance to previous therapies; and the extension and location of the disease are important.

Actinomycetoma must be treated with TS alone or in combination with other available antibiotics. Amikacin has been proven effective in disseminated infections or cases resistant to previous therapy. Renal and auditory evaluations are essential. Carbapenems are useful in some disseminated infections. Amoxacillin-clavunate can be used in some cases and during pregnancy.

Most patients with eumycetoma are treated with either ketoconazole or itraconazole. Itraconazole 200–400 mg daily for 6 months is used to create a good fibrous capsule around the lesion, followed by wide local excision, continuing itraconazole 200–400 mg daily until cure is achieved. Cure is defined by the disappearance of the mass and all sinuses, normal ultrasound, and negative mycology findings. The decision to stop therapy is determined by complete sinus healing, disappearance of the eumycetoma mass clinically and radiologically by CT scan or MRI, and absence of the infecting agent. Other antifungal agents that can be used as second-line treatment include voriconazole and posaconazole.

## Looking Forward

Actinomycetoma requires prompt diagnostic procedures to define the etiological agent. Precise identification of species by molecular techniques can achieve this goal and provide knowledge for testing the antimicrobial susceptibility patterns of each species of aerobic actinomycetes to determine the best drug regimen for clinical use. Future universal availability of these techniques in endemic areas with actinomycetoma will facilitate this objective.

There are currently no treatment guidelines for eumycetoma. Treatment is based on personal experience and a few case reports or case series. There is a pressing need to develop guidelines. The lack of prospective randomized clinical trials on fungal therapeutics makes the choice and duration of treatment of eumycetoma with antifungal agents difficult. Multicenter clinical trials to develop novel antifungals are required as current drugs are of limited efficacy, have adverse effects, and are expensive. Drug choice in eumycetoma is largely determined by availability and cost.

The International Mycetoma Center in Sudan and centers in other countries such as the Netherlands, England, Switzerland, and Mexico are joining efforts to design clinical studies to select and evaluate the best therapeutic regimes for mycetoma.

In February 2013, a meeting was convened in Geneva, supported by the Drugs for Neglected Diseases initiative (DNDi), to highlight disease awareness and propose inclusion of this infection in the list of neglected tropical diseases (NTDs) of the World Health Organization. Experts from Sudan, United Kingdom, the Netherlands, Mexico, and Switzerland participated in that event, and the Mycetoma Consortium was established. In May 2013, the proposal led by Professors Ahmed Fahal and El Sheikh Mahgoub and other researchers had a safe landing at the WHO, and by July 2013 mycetoma was included in the WHO NTDs list. This action will increase awareness and facilitate and promote international studies on new effective antifungal and antibacterial agents for the treatment of mycetoma.

**Box 1. Key Learning Points**

- Current management alternatives in the medical treatment of actinomycetoma are successful in most cases.
- Knowledge of the different antibiotic susceptibility patterns of different pathogenic species of actinomycetes is useful for selecting the best treatment.
- Combination treatments in actinomycetoma have been successful in recalcitrant cases.
- Drug adverse effects and interactions in mycetoma therapy are factors that should be considered
- Traditional and new azoles are the current potential alternatives for medical treatment of eumycetoma, but large controlled studies are lacking.

**Box 2. Top Five Papers**

1. Welsh O, Vera-Cabrera L, Welsh E, Salinas MC (2012) Actinomycetoma and advances in its treatment. Clin Dermatol 30: 372–381
2. Fahal AH, Rahman IA, El-Hassan AM, Rahman ME, Zijlstra EE (2011) The safety and efficacy of itraconazole for the treatment of patients with eumycetoma due to Madurella mycetomatis. Trans R Soc Trop Med Hyg 105: 127–132.
3. Brown-Elliott BA, Brown JM, Conville PS, Wallace RJ Jr (2006) Clinical and laboratory features of the Nocardia spp. based on current molecular taxonomy. Clin Microbiol Rev 19: 259–282.
4. van de Sande WW, Luijendijk A, Ahmed AO, Bakker-Woudenberg IA, van Belkum A (2005) Testing of the in vitro susceptibilities of Madurella mycetomatis to six antifungal agents by using the Sensititre system in comparison with a viability-based 2,3-bis(2-methoxy-4-nitro-5-sulfophenyl)-5- [(phenylamino)carbonyl]-2H-tetrazolium hydroxide (XTT) assay and a modified NCCLS method. Antimicrob Agents Chemother 49: 1364–1368.
5. De Sarro A, La Camera E, Fera MT (2008) New and investigational triazole agents for the treatment of invasive fungal infections. J Chemother 20: 661–671.
